# Argentina’s Local Crop Biotechnology Developments: Why Have They Not Reached the Market Yet?

**DOI:** 10.3389/fbioe.2020.00301

**Published:** 2020-04-09

**Authors:** Dalia Marcela Lewi, Carmen Vicién

**Affiliations:** ^1^Instituto de Genética, CICVyA, INTA, Buenos Aires, Buenos Aires, Argentina; ^2^School of Agriculture, University of Buenos Aires, Buenos Aires, Argentina

**Keywords:** regulatory system, GMO biosafety, Argentine regulation, local developments, commercial approval

## Abstract

Plant biotechnology in Argentina started at the end of the 1980s, leading to the development of numerous research groups in public institutions and, a decade later, to some local private initiatives. The numerous scientific and technological capacities existing in the country allowed the early constitution in 1991 of a sound genetically modified organisms biosafety regulatory system. The first commercial approvals began in 1996, and to date, 59 events have obtained permits to be placed on the market, however, only two have been developed locally by public-private partnerships. The transgenic events developed at public institutions pursue different objectives in diverse crops. However, once these events have been developed in laboratories, it is difficult to move toward a possible commercial approval. In this work, we analyze several reasons that could explain why local developments have not reached approvals for commercialization, highlighting aspects related to the lack of strategic vision in the institutions to focus resources on projects to develop biotechnological products. Although progress has been made in generating regulatory rules adapted to research institutes (such as the regulations for biosafety greenhouses and ways of presenting applications), researchers still do not conceive regulatory science as a discipline. They generally prefer not to be involved in the design of regulatory field trials or regulatory issues related to the evaluation of events. In that sense, some of the aspects considered a regulatory affairs platform for the public scientific system and the reinforcement of laboratories that perform tests required under the Argentine regulation.

## Brief History of Biotechnology in Argentina

Plant biotechnology in Argentina started at the end of the 1980s, leading to the development of numerous research groups in public institutions and, a decade later, to some local private initiatives. However, a prospective analysis of the local capacities of Argentina for the development and marketing of events derived from biotechnology would have led to a much more optimistic scenario than the one observed nowadays. Argentina’s experience with plant biotechnology began with pioneers such as Esteban Hopp, at the National Institute of Agricultural Technology (INTA), and Alejandro Mentaberry, at the National Scientific and Technical Research Council (CONICET), in the late 1980s, both of whom mentored the subsequent generations of specialized academics in the area.

Farmers in Argentina have always rapidly adopted new developments and technologies. Indeed, Argentine fields currently have more than 24.9 million hectares of GM crops, 19.2 of which are of soybean (almost 100%), 5.5 of maize (96%), and 0.3 of cotton (almost 100%). These data indicate that farmers are not reluctant to adopt these crops, the vast majority of which are developed abroad (ISAAA, 2018).

In 1991, the National Advisory Commission on Agricultural Biotechnology (known by the Spanish acronym, CONABIA), whose function consists of reviewing the safety assessments of biotechnology events, was formed. CONABIA is still operative today^1^ and its members include, among others, specialists in the fields of genetics, plant physiology, and agronomy. A significant aspect of the Argentine regulatory system is that it is widely recognized as being a structure that has remained “uncontaminated” by bureaucratic history, where scientific and technical credibility and enforceability prevail, which is critical when dealing with a sensitive issue for society, such as GM crops (Vicién and Trigo, 2017).

Since its creation, CONABIA has been instrumental to the successful evaluation of more than 50 different (single and stacked) events. Thanks to its outstanding academic members and excellent track record in the field, in 2014, CONABIA was recognized as a Reference Center for the Biosafety of genetically modified organisms (GMOs) by the FAO. Considering these facts, Argentina should have been much more successful in the deregulation of its local biotechnological events. However, only two out of the more than fifty events that have been approved for commercialization were developed locally.

**FIGURE 1:**
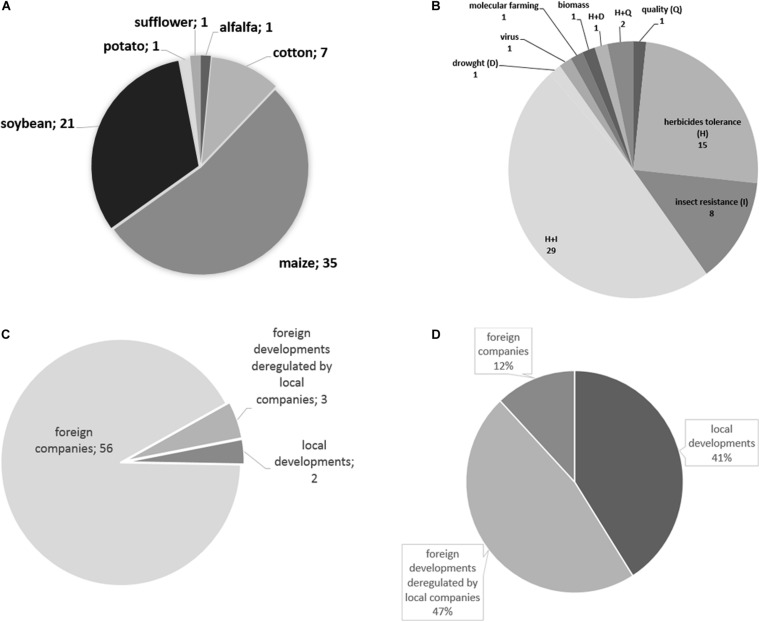
Approved biotechnological events in Argentina. Graphics made based on data published on the site https://www.argentina.gob.ar/ogm-comerciales. **(A)** number of events by crop; **(B)** number of events by trait; **(C)** number of events approved by type of developer; **(D)** cases of gene editing consulted (A. Whelan, personal communication).

The regulatory process in Argentina is established in Resolution 763/2011 issued by the Ministry of Agriculture. This Resolution establishes a procedure divided into three steps: (i) an environmental assessment performed by CONABIA, (ii) a food and feed safety evaluation performed by the National Agri-Food Health and Quality Service (known as SENASA, by its Spanish acronym), and (iii) an evaluation of its impact on the agricultural market. Once each step is completed, a Decision Document is drafted, which must be favorable for the event to be approved. The procedure is the same for both local and imported events. Most of the events that have passed through the regulatory process have been developed by private companies, mostly from the Northern Hemisphere^2^ (Figure 1: events approved in Argentina).

Table 1 shows the two national events approved for commercialization that have completed all the steps of the regulatory process: the abiotic stress-resistant and herbicide-tolerant Soybean HB4, developed by the local business company INDEAR (a public and private partnership formed by CONICET and the enterprise BIOCERES), and the virus Y-resistant Potato PVY, developed by CONICET and achieved by the company Sidus-Tecnoplant, a public-private collaboration.

**TABLE 1:** List of biotechnological developments in Argentina.

Crop	Trait	Maximum degree of progress achieved				Type of institution
			
		Greenhouse/laboratory	Field trials	CONABIA/SENASA approved	Commercial approved/deregulated	
Potato	Bacteria	×				Public
	Fungal	×				
	Quality	×				
	Nutrition facts	×				
	Virus PVX-PLRV	×	×			
	Virus PVY	×	×	×	×	Public/private
Wheat	Nutrition facts	×				Public
	Abiotic stress	×	×			
	Abiotic stress	×	×	×		Private
Alfalfa	Herbicide	×	×			Public
	Abiotic stress	×	×			
Orange	Virus	×	×			Public
Maize	Abiotic stress	×	×			Public
	Abiotic stress	×	×			Private
Cotton	Boll weavil	×				Public
Sugarcane	Virus	×	×			Private
	Herbicide	×	×	×		
Peach	Quality	×				Public
Tomato	Abiotic stress	×				Public
	Leaf area	×				
	Nutrition fatcts	×				
Lettuce	Virus	×				Public
	Fungal	×				
Sunflower	Fungal	×				Public
Grape	Abiotic and abiotic stress	×				Public
Soybean	Herbicide	×	×	×	×	Public/private
	Abiotic stress	×	×	×	×	
Paspalum	Abiotic stress	×				Public
Chloris gayana	Abiotic stress	×				Public
Fescue	Herbicide	×	×			Public

In 2015, another event was almost approved for commercialization: sugarcane with glyphosate resistance. This event passed the environmental and food and feed safety assessments, but failed to pass the agricultural market impact evaluation, possibly because of the negative public perception of sugarcane stakeholders^3^. The developments of sugarcane varieties are carried out by both public and private sector institutions.

Wheat HB4, another local development by INDEAR, which has drought resistance, is currently undergoing the evaluation and has already achieved approval from both CONABIA and SENASA, but it is still awaiting the final decision from the Agricultural Markets Office^4^. In that sense, beyond regulations, another aspect of the Argentine system that has to be considered is the internalization of potential trade problems, which is based on its position as a net exporting country. This is generally one of the main causes for the delay in approvals, since the government weighs the consequences of any new products on the Argentinian market (Vicién, 2012).

Regarding the remaining local developments listed in Table 1 (a list that may not be exhaustive), many have not gone beyond the laboratory step, others have only completed the greenhouse step, and very few have been evaluated in the field. This situation inevitably raises questions regarding the difficulties faced by local developments when looking for deregulation.

Other countries in the region are in similar situations regarding the adoption of biotechnology and are working to establish their deregulation procedures. FAO asked CONABIA to assist other countries that were establishing their regulatory frameworks, a program that is proving successful in training specialists on how to perform risk assessment for GM crops. While Argentina’s regulatory system serves as a reference for many countries, it is important to note that the system does not appear to support local developments. Fortunately, there are exceptions in the region, such as the virus-resistant beans obtained by the Brazilian Agricultural Research Corporation (EMBRAPA by its acronym in Portuguese), developed by Francisco de Aragao’s Group (Faria et al., 2016). So far, this has been the only case in which an entirely public development achieved approval by the Brazilian National Technical Biosafety Commission (CTNBIO by its acronym in Portuguese), having also completed every assay required in publicly funded labs^5^.

Over the last 30 years, the scientific-technological system in Argentina, composed of public institutions and universities, has led to the development of many GM crop events, such as potato, alfalfa, wheat, maize, sunflower, sugarcane, soybean, lettuce, and cotton (Table 1). These were achieved mostly through funding from institutions such as INTA, CONICET, Universities, and the National Agency for Scientific-Technical Promotion (ANPCyT by its acronym in Spanish) and other public sources. The projects are financially and economically evaluated taking into account research and development costs, regulatory aspects (biosecurity and varietal registration), potential benefits for farmers and for the value chain, prospective on possible markets (both internal and external), and relative sizes and possible degree of adoption. After obtaining the desired prototypes for each laboratory event, developments only reach the stage of growth chamber or greenhouse trials. However, once they are stabilized and multiplied, in many cases, there is not enough funding to advance to field trials and complete the remaining steps of the regulatory procedure.

## New Norms for New Techniques

The regulatory framework has been recently updated in Argentina. The country pioneered the development of regulations for the so-called “new breeding techniques” (NBTs), as specified in Resolution No. 173 of 2015 from the Ministry of Agriculture (Whelan and Lema, 2015) and also present in the updated version from the same Ministry (Resolution No. 36, 2019). This Resolution states that, to be considered as a “Genetically Modified Organism,” the product must possess a novel combination of genetic material obtained through the use of modern biotechnology in accordance with the definition from the Cartagena Protocol on Biosafety (Secretariat of the Convention on Biological Diversity [SCBD], 2000). Under these guidelines, the applicants must describe in detail the intended modifications and the way they plan to obtain them in an “Instance of Prior Consultation” (ICP by its Spanish acronym). CONABIA will send an answer to the applicant within 60 days to determine the regulatory status of the product. This procedure allows researchers to know whether their product will be considered as a GMO or not, even before starting laboratory work. The procedure has served as an incentive for private companies and public institutions to undertake new projects, with knowledge at the outset that costly regulatory testing will not be necessary. To date, most of the inquiries received by the authorities have come from locally developed products (Lema, 2019).

Gene editing, one of the NBTs discussed above, is one promising new biotechnological approach to improve crops that is considered more precise and can avoid the insertion of unnecessary genes. Crops modified using gene editing may be more easily adopted because products require a simpler regulatory procedure (Jones, 2015; Georges and Ray, 2017). The procedure described in Resolution No. 173/15 is streamlined for products derived from gene editing but it would still be subject to regulation, given that any product derived from the application of biotechnology is still regulated until it is determined that it does not contain stable DNA insertions. Before then, all associated material must be handled in contained and confined conditions.

## Dialogue Between Researchers and Public Regulators in Argentina

In 2014, given difficulties faced by developers in Argentina, REDBIO (a non-profit organization bringing together plant biotechnology labs across Argentina) held two workshops to debate and share ideas regarding the prevailing situation. In that sense, two official statements were drafted and addressed to the authorities responsible for regulation at the Ministry of Agriculture.

In these workshops, the various problems faced by researchers regarding the current regulatory system in Argentina were considered. The conclusions are listed below.

### Regarding Policies

  – General lack of State support for most research information and commercialization of GM events.
  – Insufficient sources of resources and State funding to go through the trials required for deregulation.
  – The non-existence of formal State structures to facilitate, organize, and present regulatory data to the relevant agencies.

### Regarding the Regulatory Process

#### For Developers

  – They are often unaware of the general regulatory process.
  – No guidelines specify how developers should start with the regulatory process.
  – The processes and steps to complete, as well as the initial data required, are unclear.
  – Many developers are not up to date on the resolutions setting out the process.

#### For the Authorities

  – There is no coordination between the agencies and offices involved in the three steps of assessment.
  – There is an overlapping of the information required from each agency.
  – Requirements and criteria are often excessive.

### Regulatory Data Generation

  – There is a need for a complete diagnosis of the available infrastructure and capabilities of the national science and technology system.
  – There is insufficient funding for regulatory studies.
  – There is insufficient information and training on the steps to follow in the regulatory process.
  – There is a need for a definition of national and international harmonized quality standards in confined field trials.

It has to be highlighted that researchers in public institutions such as INTA have always worked collaboratively with breeders, who have extensive experience in intellectual property issues, varietal registration, and the respective procedures established by the National Institute of Seeds (INASE by its acronym in Spanish).

To respond to the needs raised in the 2014 workshops, two trainings for researchers were organized by public institutions such as INTA and CONICET and NGOs such as Redbio, ILSI, and Argenbio. Researchers from all plant biotechnology development centers of Argentina participated in them and their projects were analyzed concerning how regulatory studies should be addressed.

The authorities of the Ministry of Agriculture and the Ministry of Science and Technology who participated in these workshops and acknowledged the problems discussed there created a competitive financial funding program for regulatory studies. This program was organized by ANPCyT in 2015 and was called FONREBIO (Biotechnological Products’ Regulation Funding)^6^. This funding was initially conceived as a non-reimbursable subsidy for public institutions. However, by the time of its issuance and implementation at the end of 2015, it became a sort of reimbursable loan, which required the approval for a private source of partial backing or commitment for quick insertion into the market. It was announced as a loan of ARS 20 million (USD 1 million at that time), consisting of up to 80% of the project’s total cost, with at least 20% of its funding coming from private sources. This restricted the chances of projects coming from public institutions and forced these institutions to seek support from companies interested in investing in the projects. Furthermore, the chances for a project to advance were tied to its intrinsic potential for commercial success.

Similarly, INTA established an internal contest for the use of royalty funds owned by the institution (known as “*Fondos de valorización tecnológica*”). The funding was meant to promote the insertion of products developed by INTA, including biotech events, into the market. To apply, the development must be commercially viable and easily adopted by producers, which again limits the availability of funding to economically competitive developments only. At the beginning of 2018, the funding consisted of ARS 20 million (USD 1 million back then but only USD 300 thousand nowadays due to the devaluation of the Argentine peso) directed at projects of many different origins, including biotech events. The amount offered quickly proved to be insufficient. Once again, an initiative to boost local developments ended up being inadequate to achieve that goal.

As previously mentioned, local projects can have many different goals. Some of them focus on productivity, which gives them better chances to compete for funding to pay for regulatory studies successfully, whereas others are meant to enhance quality, or are directed at small producers or family farms, which leaves their chances to acquire private backing limited or completely cut off. This means that they end up being unable to pay for the cost of the regulatory process, thus keeping the product from ever reaching the market.

Regarding the costs of the deregulation process, some estimates indicate that it is roughly ten times that of the development itself. These estimates include the costs of gene discovery together with those of the deregulation in various countries, which is partly why the total amount needed varies so much (Kent, 2004; Falck-Zepeda et al., 2012; Prado et al., 2014). Variation in cost depends on the number of countries where it is filed and the nature of the studies required according to the approval policies of each country. Even though in most cases the necessary studies to achieve approval for commercialization are well established, the amount of money needed to complete them is up for debate, and can be high enough for small businesses or public institutions to abandon or delay the marketing of locally potentially valuable products. Currently, mainly private multinational companies can afford the regulatory burden of approvals in different countries.

Another factor that makes this process even more expensive is the quality certification needed for the data obtained in the regulatory studies. For the EU, the data required must be GLP (Good Laboratory Practices) certified. This certification is handled by the Argentine Accreditation Body (OAA by its Spanish acronym). So far, only a few institutions have been able to achieve this certification, which is expensive. SENASA and CONABIA do not currently require this certification but procedures and data integrity are thoroughly examined.

The current state of event approval across the globe must also be taken into consideration. To avoid problems with imports and exports, the approval of a single event is often requested in more than one country at the same time (for example, a maize event may be presented simultaneously in the United States, European Union, Brazil, Argentina, Colombia, and Paraguay). Every country added to that list results in additional costs and resources. To facilitate the process for their products, many international companies engage their own Regulatory Affairs Departments. This practice allows an efficient organization of both human and financial resources. Until now, there is nothing similar in public institutions.

Based on all the above, the following questions can be posed: Can a local project achieve deregulation in the country? Can it achieve the same acceptable standards for safety as a previously approved privately funded one? Can locally funded projects afford de-regulation in other countries?

## The Paradox

As a result of the foregoing, public institutions find it almost impossible to raise funds for the deregulation process, the cost of which exceeds any funding that may be obtained. Thus, a paradox is established, whereby products derived from plant biotechnology, such as GMOs, are developed to address production problems and improve crop quality in ways that conventional breeding cannot achieve, and yet, for these products to be approved and thus be used by farmers, they must meet the criteria set by regulatory authorities in each country where they are expected to be commercialized. The required regulatory studies are extraordinarily expensive and can only be paid for if the institution or company where the product was developed has the necessary funding.

Consequently, only private developments with sufficient funding and adequate resource management are able to pay for the required studies and their certification. Therefore, the only products to ever reach the market are those derived from private initiatives of transnational firms. Developments seeking to solve problems for local production or small farmers are seldom given a chance to reach approval, as they are less attractive for large companies because markets and sales are smaller in size. Furthermore, even when they are granted approval, after much effort and search for company help, as it happened with PVY-resistant potato, developers may find obstacles to commercialization because of public perception barriers. Another controversial case is that of wheat HB4^®^, which was approved by two out of three agencies (CONABIA and SENASA) but rejected by the agricultural market evaluation, because of the unfavorable public perception of the crop’s value chain.

In this context, further questions that can be posed include: How can this problem be solved? Which institutional paths can be explored? What should be proposed?

As mentioned before, private multinational companies have regulatory affairs departments, composed of professionals in charge of designing the studies, managing agency permits, performing field trials, and conducting follow-up of developments to comply with regulatory criteria all over the world. Public institutions do not have anything like that. A good start for public policies meant to help the development of local biotechnology through the organization of human and financial resources would be the creation of a government agency for GMO regulatory affairs as a shared platform to make the process faster, easier, and more efficient. Local researchers and developers must also consider starting a dialogue with every participant in the value chain, including producers and coordinators of crop breeding programs, from the very beginning of the initial development (the original idea). These considerations can be applied to GMOs, as well as to products obtained through the application of gene editing and other new tools of biotechnology.

## Data Availability Statement

Publicly available datasets were analyzed in this study. This data can be found here: https://www.argentina.gob.ar/agricultura/alimentos-y-bioeconomia/ogm-comerciales.

## Author Contributions

Both authors participated in the drafting of this manuscript as individual experts in their fields, and are solely responsible for the contents. Any views expressed in this manuscript are the views of the authors and do not necessarily represent the views of any organization, institution, or government to which they are affiliated or employed.

## Conflict of Interest

The authors declare that the research was conducted in the absence of any commercial or financial relationships that could be construed as a potential conflict of interest.

## Footnotes

1 https://www.argentina.gob.ar/convocatoria-conabia

2 https://www.argentina.gob.ar/ogm-comerciales

3 http://www.sitioandino.com.ar/n/176588-ingenios-se-resisten-a-la-cana-de-azucar-transgenica/

4 https://www.clarin.com/economia/economia/gen-resistencia-sequia-divide-aguas-gabinete-macri.html

5 http://ctnbio.mctic.gov.br/documents/566529/686210/Parecer+Consolidado+Francisco+Zerbine.pdf/2217911a-07fb-4643-8757-e9badcbe5516?version=1.0

6 http://www.mincyt.gob.ar/convocatoria/fonrebio-11901
